# Coupling Developmental Physiology, Photoperiod, and Temperature to Model Phenology and Dynamics of an Invasive Heteropteran, *Halyomorpha halys*

**DOI:** 10.3389/fphys.2016.00165

**Published:** 2016-05-18

**Authors:** Anne L. Nielsen, Shi Chen, Shelby J. Fleischer

**Affiliations:** ^1^Department of Entomology, Rutgers UniversityBridgeton, NJ, USA; ^2^Department of Population Health and Pathobiology, North Carolina State UniversityRaleigh, NC, USA; ^3^Department of Entomology, Pennsylvania State UniversityUniversity Park, PA, USA

**Keywords:** brown marmorated stink bug, phenology, agent-based model, stochastic model, life-history, population dynamics, invasive species

## Abstract

We developed an agent-based stochastic model expressing stage-specific phenology and population dynamics for an insect species across geographic regions. We used the invasive pentatomid, *Halyomorpha halys*, as the model organism because gaps in knowledge exist regarding its developmental physiology, it is expanding its global distribution, and it is of significant economic importance. Model predictions were compared against field observations over 3 years, and the parameter set that enables the largest population growth was applied to eight locations over 10 years, capturing the variation in temperature and photoperiod profiles of significant horticultural crop production that could be affected by *H. halys* in the US. As a species that overwinters as adults, critical photoperiod significantly impacted *H. halys* seasonality and population size through its influence on diapause termination and induction, and this may impact other insects with similar life-histories. Photoperiod and temperature interactions influenced life stage synchrony among years, resulting in an order of magnitude difference, for occurrence of key life stages. At all locations, there was a high degree of overlap among life stages and generation. Although all populations produced F_2_ adults and thus could be characterized as bivoltine, the size and relative contribution of each generation to the total, or overwintering, adult population also varied dramatically. In about half of the years in two locations (Geneva, NY and Salem, OR), F_1_ adults comprised half or more of the adult population at the end of the year. Yearly degree-day accumulation was a significant covariate influencing variation in population growth, and average maximum adult population size varied by 10-fold among locations. Average final population growth was positive (Asheville, NC, Homestead, FL, Davis, CA) or marginal (Geneva, NY, Bridgeton, NJ, Salem, OR, Riverside, CA), but was negative in one location (Wenatchee WA) due to cooler temperatures coupled with timing of vitellogenesis of F_2_ adults. Years of the highest population growth in the mid-Atlantic site coincided with years of highest crop damage reports. We discuss these results with respect to assumptions and critical knowledge gaps, the ability to realistically model phenology of species with strongly overlapping life stage and which diapause as adults.

## Introduction

Phenology modeling of insects typically relies on temperature-dependent functions that describe development as the transition among immature life stages and into the adult life stage. Insect development is often expressed in terms of degree-days (DD), which can be modeled to time management applications, can determine the number of generations across locations, and can be integral in invasive species risk assessments (Damos and Savopoulou-Soultani, 2012). However, DD models ignore life-history functions that are not primarily influenced by temperature, such as the transition between diapausing and non-diapausing life stages. This constrains the utility of single parameter models for insects that overwinter as diapausing adults, where a biofix to initiate DD models is frequently unclear. When used to time management inputs or predict life cycle events in agricultural and forest systems, DD models compensate by establishing a biofix that defines an initial time where development among a subset of life stages, all of which are strongly influenced by temperature, can be initiated. For example, capture of adults in traps is generally used to imply activity including mating or oviposition, which is then used to initiate DD models to estimate the time for development of eggs to more advanced, damaging life stages (Riedl et al., 1976; Wolda, 1988). While this approach has been successful to time management inputs, it limits the scale to the area where this biofix is measured, and requires the ability to define a biofix, often with field measurements.

A variety of insect phenology models have been developed that enable estimation of both diapausing and non-diapausing life stages across wide spatial scales (Gray, 2004; Logan et al., 2007; Powell and Bentz, 2009; Bentz et al., 2014). Phenology models can incorporate physiological variation and additional parameters; one method is through application of agent-based modeling which has been successfully applied to lepidopterans to express the expected distributions of each life stage (Chen et al., 2011; Nealis and Régnière, 2014). Such agent-based methods better approximate realism for a species that has strongly overlapping life stages and heterogeneity in population traits (e.g., egg clutch size, mortality rate, etc.). This approach also enables modeling the distributions of diapausing and non-diapausing adults, which could better define the voltinism potential of insects that overwinter in the adult stage, and effectively captures the interaction of explanatory variables (i.e., photoperiod and temperature) on both development and population dynamics.

We use *Halyomorpha halys* (Stål) (Hemiptera: Pentatomidae) as an model organism to evaluate a stochastic agent-based model where purely temperature-based models might not be appropriate because it overwinters in adult diapause, has strongly overlapping generations, and sampling methods for defining a field biofix are unclear (Nielsen and Hamilton, 2009). As for most temperate insect species, diapause termination, and initiation cues which are influenced by genetics and metabolism as well as extrinsic factors such as temperature and photoperiod (Tauber et al., 1986), have yet to well defined for *H. halys*. Short-day photoperiod as the diapause cue has been identified in many species (reviewed in Koštál, 2011). Utilizing a “standardized” cue such as photoperiod allows insects to keep track of time similar to a circadian clock and evidence is mounting that physiological development post the critical photoperiod is genetically controlled (Bradshaw and Holzapfel, 2010; Koštál, 2011). Species that do so as adults are frequently in a reproductively immature state, which could serve as a physiological adaptation to reduce energy needs during diapause (Numata and Hidaka, 1982; Saunders, 1983; Saunders et al., 1989; Santos et al., 2003). We cautiously assume that diapause termination and induction cues for *H. halys* are the same and are triggered by photoperiod, independent of temperature (Watanabe, 1979; Yanagi and Hagihara, 1980).

As with many other invasives, *H. halys* is a pest that is rapidly expanding its geographic range and pest status in North America and Europe and outbreak densities have caused significant economic loss (Leskey et al., 2012; Rice et al., 2014; Haye et al., 2015; Lee, 2015) and climate niche modeling suggests additional potential expansion (Zhu et al., 2012). *H. halys* overwinters as non-feeding, adults in aggregations on cliff outcroppings, dead upright trees, or human-made structures (Watanabe et al., 1994; Lee et al., 2014) in a non-reproductive (i.e., diapausing) state. The exhibit a gradual emergence from overwintering sites and field estimates on the timing of transition to non-diapausing development and voltinism is difficult due to overlapping generations and a broad host range (Nielsen and Hamilton, 2009; Nielsen unpub.). *H. halys* goes through the egg stage plus five developmental instars before the final molt to adult. Overwintering (diapausing) female *H. halys* are pre-vitellogenic (Nielsen, unpublished), and require an additional period of development prior to sexual maturity. Watanabe (1979) suggests that the critical photoperiod for ovarian development for *H. halys* is between 13.5 and 14.0 h in Toyama, Japan. In temperate latitudes, overwintering *H. halys* adults retain a diapausing physiological state well into the spring before initiation of reproduction (Nielsen, unpublished). Non-diapausing females continue to reproduce throughout their lifespan, which can last up to a few months post diapause emergence (Nielsen et al., 2008; Haye et al., 2014). Other life history components of *H. halys* including pre-ovipositional period, fecundity, nymphal development, and survivorship have been described in relation to temperature for populations in Asia and the US (Yanagi and Hagihara, 1980; Nielsen et al., 2008; Nielsen, unpublished). Although these life table measures are defined in relation to abiotic conditions—and thus do not capture effects due to biotic factors (predation, parasitization, nutrition, etc.)—they provide a basis for modeling the phenology of *H. halys* across a landscape, and for varying landscapes in which this species has recently invaded.

Our objective is to develop a model to estimate voltinism, stage-specific phenology, and population dynamics of an insect with unclear developmental requirements. We do this using *H. halys* as a model organism and predict population dynamics at agriculturally relevant locations across the continental US including locations prior to economically damaging populations. We develop an agent-based model as a synthesis of existing information about *H. halys* life history and demography (e.g., birth and death) as influenced by photoperiod and temperature, and compare model outputs with field observations. We use these validation efforts, along with varying specific photoperiod sensitivity for diapause induction and termination, to focus attention to areas in need of further study by demonstrating sensitivity of simple population dynamics models to these parameters defining physiology. We then apply the most effective models to eight locations in the US that are relevant to horticultural crops at risk of damage over a 10 year timeframe, which span the latitudes of recent and potential invasion in the US and current climatic conditions.

## Methods

### Model formulation

**Figure 1:**
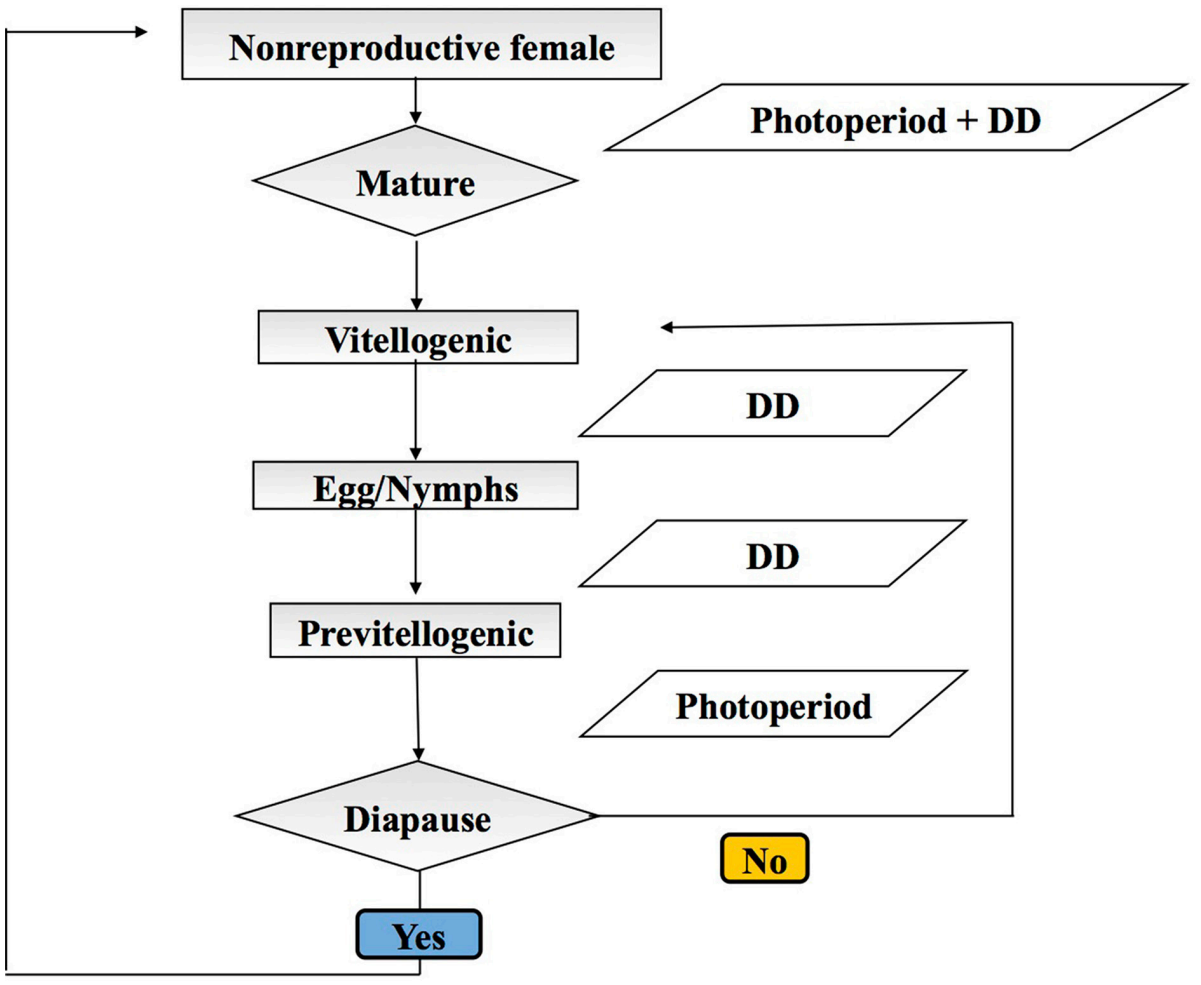
**Diagram of the *Halyomorpha halys* individual-based model steps during development**. Squares indicate developmental stages and parallelogram indicate inputs. Diapause is represented by a diamond and represents a key physiological decision point.

We adopt an agent-based stochastic modeling framework to explicitly track the life history and population dynamics (through birth and death) of *H. halys* (see Grimm et al., 2006). Each model run is initiated on January 1 with 1000 overwintering female *H. halys*, all categorized as pre-vitellogenic diapausing adults, and continues until the end of the year when the successive generations' adults are in diapause. Each individual *H. halys* is an autonomous agent in this model, and they develop, reproduce, and die according to the environmental factors (temperature and photoperiod). The initial adults are considered the parent generation (P). The model comprises five major modules: (i) diapause termination of generation P, (ii) fecundity of P and successive generations of adults (F_1_, F_2_, …F_x_), (iii) development from egg to adult, (iv) diapause induction of successive (F_x_) adults, and (v) survivorship in all generations (Figure 1). Among these five modules, diapause termination and induction are photoperiod-mediated (Yanagi and Hagihara, 1980), while development and survivorship are primarily dependent on temperature (Nielsen et al., 2008). Reproduction is assumed to be mostly environmentally independent, although the newly eclosed adult females need to accumulate about 68 degree-days using a lowest development threshold temperature of 12.7°C (denoted as DD_12.7_) prior to oviposition (Yanagi and Hagihara, 1980). The development module is divided into six stages with varying responses to temperature: from egg to 1st instar nymph (egg incubation), between two consecutive nymphal instars (nymphal development, four processes in total), and between the last (5th instar) nymph and adult (eclosion). We use the temperature-dependent development rates reported by Nielsen et al. (2008), and linear relationships between temperature and development rate for each process computed from these published data (Table 1). In each day, each individual *H. halys* has a specific development rate based on temperature and survivorship (Nielsen et al., 2008), and this rate is further applied in a Bernoulli trial to simulate whether the individual will develop to the next stage, and this step imparts stochasticity. For example, two individuals that have exactly the same development rate may result in a different development result (i.e., whether or not transit to next life stage) because of the outcome of the Bernoulli trial. We also use the minimum and maximum developmental thresholds estimated from Nielsen et al. (2008), as 14.17°C and 35.76°C, respectively. Beyond this temperature range, development is halted. In addition, the mean degree-day requirements for egg incubation and complete life history are 53.30 DD_13.94_ and 537.63 DD_14.14_, respectively (Nielsen et al., 2008).

**Table 1:** **Stage-specific development rate as function of daily mean temperature**.

**Process**	**Life stage**	**Development rate (*y* = f(T))**	***R*^2^**
Egg incubation	Egg–1st instar	*y* = −0.9843T + 33.438	0.88
Development	1st instar–2nd instar	*y* = −0.3728T + 14.68	0.84
	2nd instar–3rd instar	*y* = −0.6119T + 25.249	0.81
	3rd instar–4th instar	*y* = −0.3986T + 17.602	0.77
	4th instar–5th instar	*y* = −0.4408T + 19.036	0.82
Emergence	5th instar–Adult	*y* = −0.5332T + 24.147	0.90

*P < 0.01 for all life stages*.

*T, daily mean temperature (°C); y, percent development per day for transition through that life stage; e.g., if y = 0.2 then the individual has 20% probability of transition to the next life stage*.

The survivorship module consists of three submodules: for immatures (egg and nymphal stages), for overwintering adults (P), and for all the successive generation adults (F_x_). The temperature-dependent immature survivorship rates are reported in Table 2 from Nielsen et al. (2008) and we assume a 1:1 sex ratio. Overwintering adult (P generation) mortality rates post-diapause termination was extracted from Haye et al. (2014). This step also involves stochasticity similar to the development module because of the stochasticity of Bernoulli trial. In addition, eggs and nymphs will die once the daily minimum temperature falls below 0°C after the autumnal equinox. We also make the conservative estimate that reproductive state is not plastic, and thus adults who are reproductively mature (“vitellogenic”) prior to the diapause initiation cue of critical photoperiod will not enter a diapausing state, and with all other vitellogenic adults will die at 0°C. In the fecundity module, a total of 68 DD_12.7_ is required for the pre-oviposition period (Yanagi and Hagihara, 1980). Clutch size (number of eggs per oviposition, mean ± sem) is 26.08 ± 0.31, the interval between clutches is 4.32 ± 0.41 days, the number of oviposition events is 9.33 ± 0.19, and the number of hatched eggs per clutch are 21.30 ± 0.48, as reported in Nielsen et al. (2008). Each individual vitellogenic female is simulated with a specific oviposition event number, clutch size, and number of hatched eggs (all rounded to the nearest integer). The sex ratio is 1:1.

The model ran for 100 simulations (replication) per site per year (from Jan. 1st to Dec. 31st) to account for stochasticity associated within all the module/submodules. At the beginning of each simulation (Jan. 1st), 1000 overwintering pre-vitellogenic adults were introduced. The model ran at a discrete daily step to check the potential mortality, fecundity, and developmental transition for each individual. The model also kept track of each individual's life stage or phenological category (e.g., whether an individual was in P or F_x_ generation); and in each specific nymphal and adult reproductive state, and added new births and removed dead individuals to the population. At the end of each simulation, metrics such as daily population size, mean eclosion date for each generation, and degree-day accumulation were collected for further analysis. The model started fresh for a new year (i.e., with 1000 overwintering adults) instead of carrying over from the previous year. All scripts (including all the modules described previously) were written and verified in *R* (v. 3.1.0).

### Influence of photoperiod

The critical photoperiod for diapause termination for *H. halys* is reported to range from 13.5 to 14.75 h (Watanabe, 1979; Yanagi and Hagihara, 1980). We bracketed a critical photoperiod range from 13.5 to 14.5 h, with a 0.5 h step, and compared *H. halys* population dynamics in these three conditions. For simplification, we assume that this critical photoperiod is universal for all individuals in the population (e.g., no individual variation of critical photoperiod cue), and the critical photoperiod for diapause termination prior to the summer solstice is the same as critical photoperiod for diapause induction following the summer solstice. Furthermore, the photoperiod experienced by the F_x_ adult on the first day of eclosion determines the diapause category for that female. In contrast to the F_x_ adults, who remain in diapause once they are placed in that category, P adults transition from diapausing to vitellogenic once the critical photoperiod prior to the summer solstice is reached. We chose Allentown, PA (40.40°N, 75.48°W) in 2007 to evaluate the influence of photoperiod by comparing the total and adult population size for the F_1_ and F_2_ generations under the three photoperiods (13.5, 14.0, and 14.5 h) from 100 simulations using analysis of variance (ANOVA). For model validation and prediction of voltinism across geographic locations we selected the critical photoperiod that resulted in the highest population size as determined by a one-way ANOVA.

### Model validation

Model predictions for Allentown, PA (40.40°N, 75.48°W) were validated using seasonality and density estimates collected with beat sheets from ornamental plants in Allentown, PA, for the years 2005–2007 (see Nielsen and Hamilton, 2009). Life stage-specific numbers of *H. halys* collected twice-weekly were summed across host plants. For validation the population sizes for both observed and predicted populations were scaled relative to the maximum observed population size observed each year and plotted accordingly for comparison. Coefficients of determination (*R*^2^) were computed between field observations and model predictions for three life stages (young nymph, old nymph, and adult) in 3 years (2005–2007).

### Phenology and dynamics at eight locations

Subsequent model runs were conducted for the following eight locations representing current and potential areas of *H. halys* invasion across the continental US: Wenatchee, WA (47.42°N, 102.33°W), Salem, OR (44.93°N, 123.03°W), Davis, CA (38.55°N, 121.74°W), Riverside, CA (33.95°N, 117.40°W), Geneva, NY (42.88°N, 76.99°W), Bridgeton, NJ (39.43°N, 75.23°W), Asheville, NC (35.58°N, 85.56°W), and Homestead, FL (25.47°N, 80.47°W). These locations were chosen because of variation in temperature and photoperiod profiles and because they represent locations of significant horticultural crop production that could be affected by *H. halys* in the US. The daily maximum and minimum temperature data were acquired from National Oceanic and Atmospheric Administration (NOAA), and the photoperiod profiles were computed based latitude and day of year following Forsythe et al. (1995). These model runs predicting seasonality and voltinism for these eight locations were conducted for a period of 10 years, from 2005 to 2014, which encompasses current climate conditions and the time period in which *H. halys* was present in most locations (Leskey et al., 2012; www.stopbmsb.org). This time frame also includes 2010, the year in which *H. halys* populations were very large in the mid-Atlantic region, and years in which temperature extremes occurred. At the end of model simulations for a given location and year, metrics such as mean number of generations, mean and variability of emergence time of various life stages for each generation, and population size (of total individuals and adults), were collected.

The influence of model predictions on population size was analyzed for maximum adult population, final population size (adults entering overwintering diapause) and total DD accumulation through the entire year. We tested for differences in population sizes among locations with analysis of covariance (ANCOVA) in *R* (ver. 3.1.0), assuming results in different years were independent observations for the same location and using year as a covariate, followed by a Tukey's HSD test to explicitly compare sites.

## Results

### Influence of photoperiod

**Figure 2:**
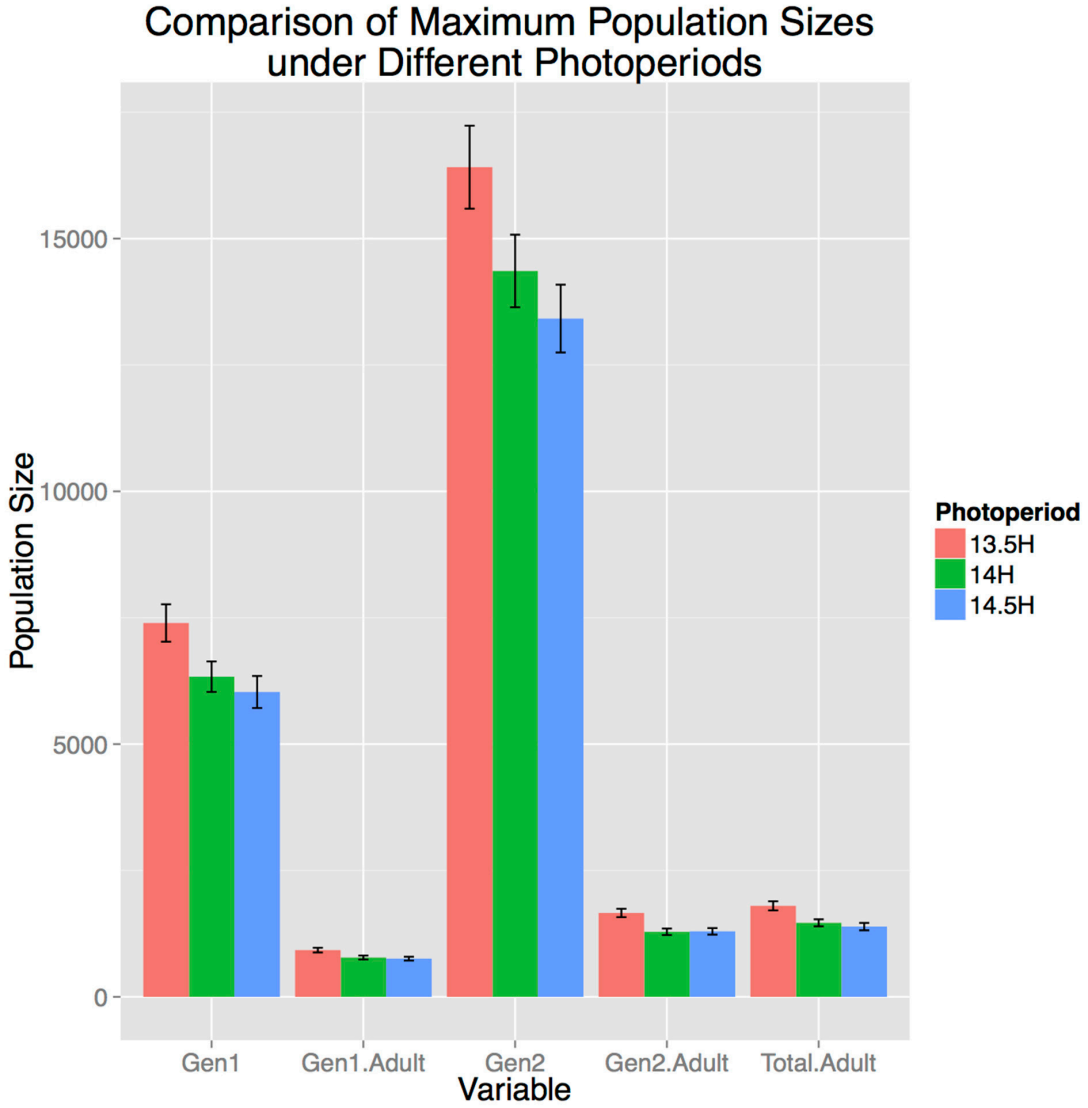
**Influence of three photoperiod cues on total population size in Allentown, PA, 2007**. Photoperiod units are hours of daylight, and are assumed to influence both diapause termination in the Spring, and diapause initiation after the summer solstice.

Photoperiod strongly influenced population dynamics and abundance. Population development in Allentown, PA occurred between April 20–August 23, May 1–August 10, and May 17–July 26, for the critical photoperiods of 13.5, 14, and 14.5 h, respectively. The 1 h difference in critical photoperiod resulted in almost a 2 month shorter time frame during which reproduction and development could occur. Consequently, shorter photoperiod cues resulted in significantly larger total population size and adult population size in both generations and total population entering diapause (*p* < 0.01, Figure 2). The most substantial difference was observed for the F_2_ adult population where a 13.5 h photoperiod resulted in 22 and 20% larger population size than a 14.0 or 14.5 h photoperiod, respectively. Furthermore, more southern locations (e.g., Homestead, FL) did not have a photoperiod ≥14.0 h and thus a critical photoperiod beyond that was not appropriate for these locations. Among the ranges reported in the literature, 13.5 h of daylength resulted in the largest populations, and thus was the most conservative estimate for considering population growth potential across the entire range of continental US. Therefore, for this study we used a 13.5 h critical photoperiod in the remaining model runs.

### Model validation

**Figure 3:**
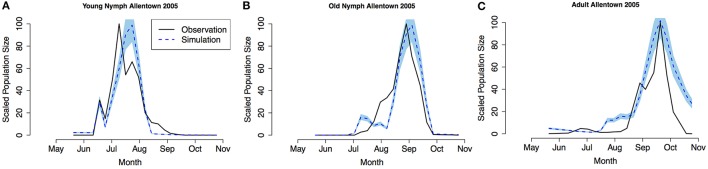
**Model validation in Allentown, PA 2005 (A) young nymphs (1st–3rd instar), (B) old nymphs (4th96-5th instar), (C) adult**. Shaded area represents 95% CI (confidence interval) for predictions from 100 simulations.

**Figure 4:**
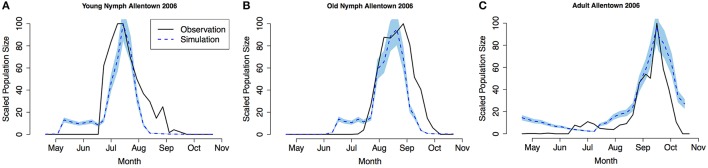
**Model validation in Allentown, PA 2006 (A) young nymphs (1st–3rd instar), (B) old nymphs (4th–5th instar), (C) adult**. Shaded area represents 95% CI.

**Figure 5:**
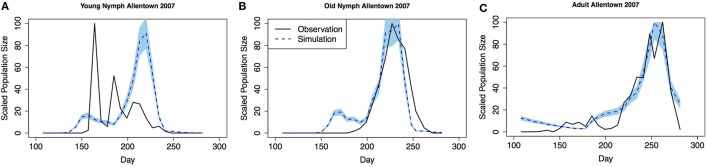
**Model validation in Allentown, PA 2007 (A) young nymphs (1st–3rd instar), (B) old nymphs (4th–5th instar), (C) adult**. Shaded area represents 95% CI.

Model validation was performed using occurrence data collected in Allentown, PA from 2005 to 2007 on ornamental trees and shrubs of various origin (Figures 3–**5**, see Nielsen and Hamilton, 2009 for data). Population phenology was divided into three life stages, young nymphs (1st–3rd instar), old nymphs (4th and 5th instar), and adults. Model outputs in all 3 years indicate that adult P, F_1_, and F_2_ generations had overlap although some distinction was observed between young and old nymph stages. In 2005, the predicted population phenology for all three life stages aligned closely with the observed phenology, especially in the timing of the largest population peaks (Figure 3). In 2006 for all life stages, and 2007 for large nymphs and adults, the timing of the largest population sizes for all three life stages also aligned closely with the field observations (Figures 4, 5). However, modeled estimates of the timing of populations in the early season were inconsistent. In 2006 for all three life stages, and in 2007 for old nymphs and adults, modeled output predicted higher values earlier in the season. Conversely, in 2007, the model did not capture field estimates of an early season spike in young nymphs. The *R*^2^ values ranged between 0.82 and 0.96 (except young nymphs in 2007 where the *R*^2^ was 0.35) for various life stages in 3 years, showing a robust prediction from the model output comparing to the field observations.

### Phenology and dynamics at eight locations

**Figure 6:**
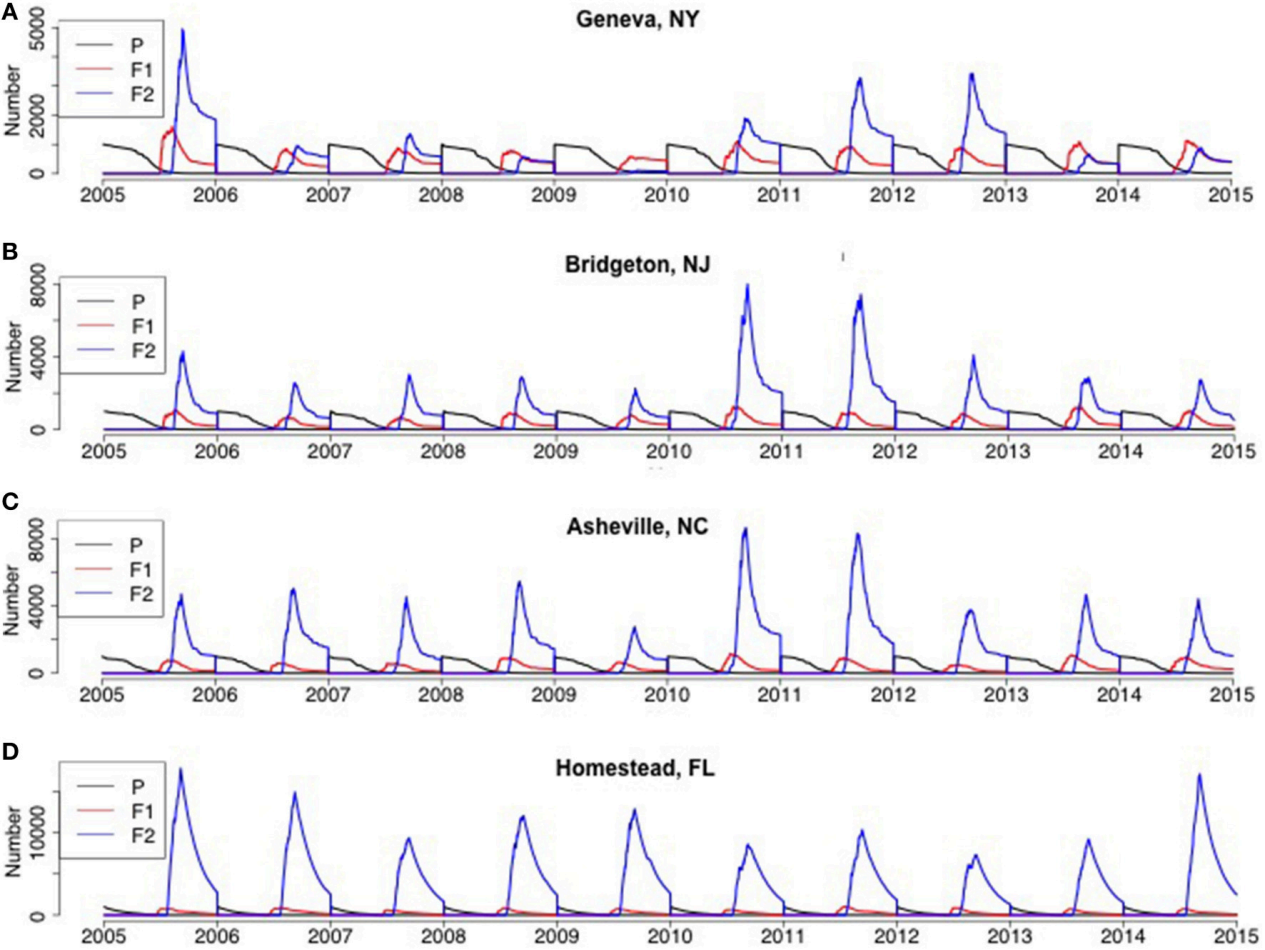
**Model predictions of adult population size by generation for the Eastern US (A) Geneva, NY, (B) Bridgeton, NJ, (C) Asheville, NC, (D) Homestead FL from 2005 to 2014**. P represented parental overwintered adults, which was initialized as 1000 each year and for each simulation run.

**Figure 7:**
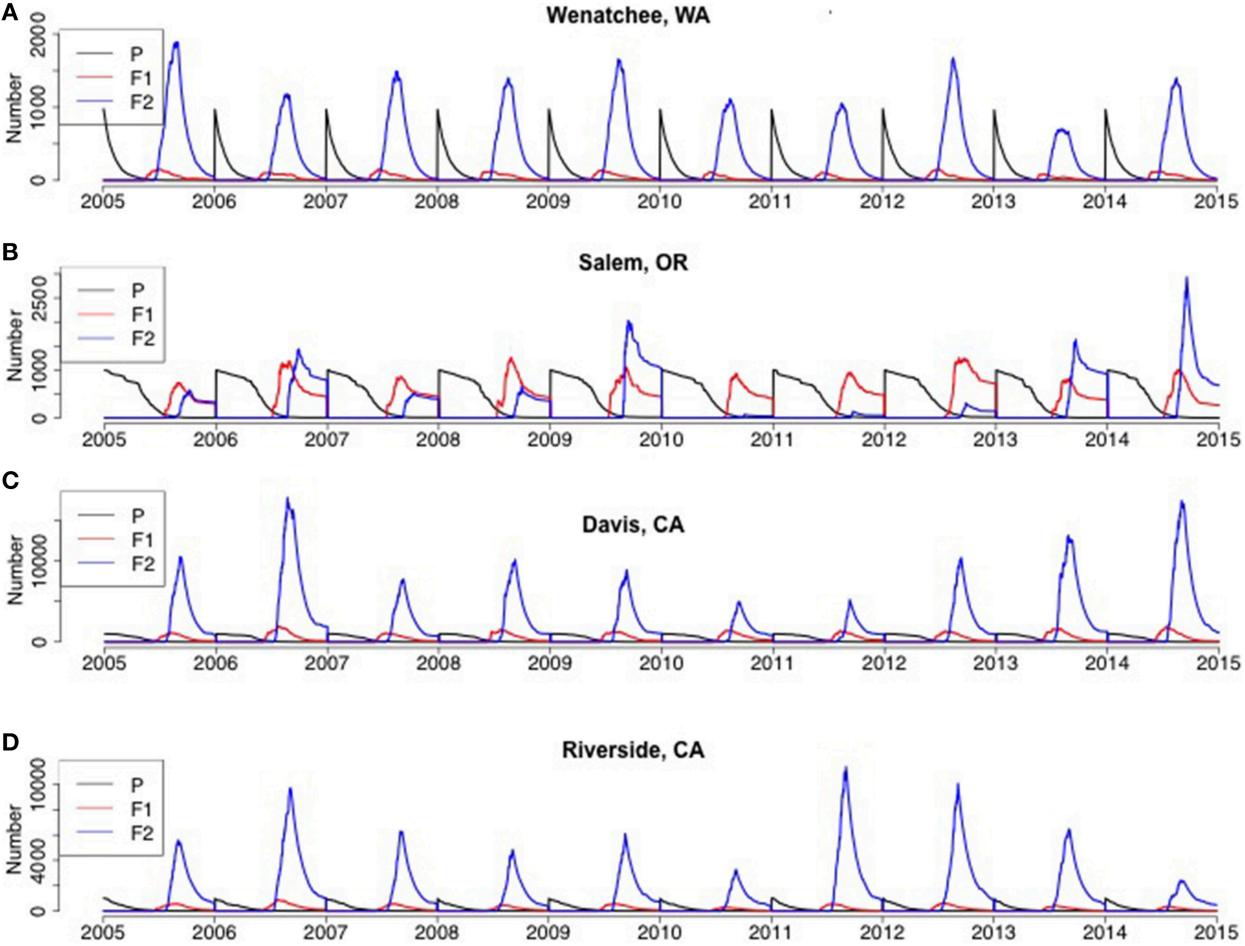
**Model predictions of adult population size by generation for the Western US (A) Wenatchee, WA, (B) Salem, OR, (C) Davis, CA, (D) Riverside, CA from 2005 to 2014**. *P* represented parental overwintered adults, which was initialized as 1000 each year and for each simulation run.

*H. halys* adults emerged from diapause in a pre-vitellogenic status (Figures 6, 7, Figure S1). The timing of diapause termination (noted as the earliest date of entering a non-diapause condition) ranged from April 14 to May 24 (Table 2) with the earliest dates being from April 14–18 at latitudes 42.88°–47.92°N. The latest diapause termination date is expected to be May 24 in Homestead, FL. Diapause termination followed a North to South gradient and diapause initiation followed a South to North gradient as would be expected for a short-day diapausing insect species. This invariant response of diapause to photoperiod strongly influenced population phenology and dynamics, as discussed below, and thus model assumptions tied to diapause represents a key knowledge gap.

**Table 2:** **Model outputs defining key population parameters for the years 2005–2014**.

**Location**	**Coordinates**	**Crop**	**Non-diapause range**	**P Oviposition**		**F**_**1**_ **Eclosion**		**F**_**2**_ **Eclosion**		**Final adult population**	
				**Range**	**Median**	**Range**	**Median**	**Range**	**Median**	**F_1_**	**F_2_**
Geneva NY	42.88°N 76.99°W	Apple	Apr 18–Aug 26	May 30–Jun 17	Jun 7	Jun 21–Jul 18	Jun 30	Jul 19–Aug 9	Jul 28	243–447	81–1847
Bridgeton NJ	39.43°N 75.23°W	Peach/Vegetable	Apr 22–Aug 22	Jun 3–Jun 6	Jun 4	Jun 9–Jul 2	Jun 23	Jul 6–Jul 31	Jul 24	140–278	531–2027
Asheville NC	35.58°N 85.56°W	Tree fruit/Vegetable	Apr 28–Aug 17	May 28–May 29	May 29	Jun 3–Jun 11	Jun 6	Jun 25–Jul 4	Jul 2	89–253	803–2287
Homestead FL	25.47°N 80.47°W	Tomato/Strawberry	May 24–July 22	Jun 5–Jun 6	Jun 6	Jun 17–Jun 18	Jun 18	Jul 8–Jul 10	Jul 9	44–137	1318–2781
Wenatchee WA	47.42°N 102.33°W	Apple/Pear	Apr 14–Aug 31	Jun 7–Jun 23	Jun 10	Jun 11–Jul 4	Jun 30	Jul 18–Aug 7	Jul 22	0–7	13–43
Salem OR	44.93°N 123.03°W	Tree fruit/Wine grape	Apr 17–Aug 28	Jun 8–Jun 9	Jun 9	Jun 17–Jul 2	Jun 26	Jul 24–Jul 31	Jul 27	269–716	33–1035
Davis CA	38.55°N 121.74°W	Tomato	Apr 24–Aug 20	Jun 3–Jun 4	Jun 4	Jun 14–Jun 19	Jun 16	Jul 6–Jul 15	Jul 11	75–255	733–1893
Riverside CA	33.95°N 117.40°W	Citrus	May 1–Aug 13	Jun 10–Jun 11	Jun 11	Jun 20–Jun 24	Jun 23	Jul 8–Jul 16	Jul 13	21–95	349–962

Initial oviposition (P oviposition in Table 2) occurred rapidly once the conditions were met, and narrowed the variation in population phenology among locations and years. Whereas, the initiation of vitellogenesis occurred over a 40 d span among locations, parental oviposition was initiated over a 26 d time span (between May 28 and June 23 among locations). Thus, median P generation oviposition was estimated to occur within a 2 week time span (May 29–June 11 among all locations and years). However, variation in temperature conditions among locations after critical photoperiod resulted in a bimodal pattern of the range over which oviposition by P generation adults occurred: most locations initiated oviposition during a rapid (1–3 d) time frame, whereas the two northernmost locations initiated oviposition over a 16 d (Wenatchee, WA) or 18 d (Geneva, NY) time span.

Development of the five immature life stages was directly influenced by temperature and resulted in eclosion of the F_1_ generation across a narrow temporal distribution in some locations, but much wider range in others. For example, F_1_ eclosion occurred between June 14–June 19 (5 days) in Davis, CA, and June 9–July 2 (23 d) in Bridgeton, NJ, despite being initiated at a similar time frame and being at similar latitudes Figures S6, S7. Although these differences among locations narrowed as the season progresses, they persisted, and result in a 9 d time span for F_2_ eclosion in Davis CA, compared to a 25 d time span for F2 eclosion in Bridgeton NJ (Table 2, Figures S2–S5).

At all geographic locations, F_2_ adults were predicted to occur and enter into diapause, thus populations at all locations could be characterized as bivoltine. Adults, male or female, that became reproductively mature prior to experiencing the diapause initiation cue were killed upon the first incidence of frost, as were all developing nymphs. However, depending on the timing of eclosion, some F1 adults became reproductive and others were conditioned for diapause. some F_1_ adults also eclosed after the critical photoperiod following the summer equinox, and thus there was a mix of both F_1_ and F_2_ adults entering diapause at the end of the year (Figures 6, 7). The relative contribution of each generation adults to the total, or overwintering, population varied dramatically across the US. For example, in five locations (Asheville, NC, Bridgeton, NJ, Homestead, FL, Davis, CA, Riverside, CA) F_2_ adults typically comprised the overwhelming majority of the adults at the end of the year. In contrast, F_1_ adults comprised a large fraction of the population at the end of the year in multiple years in Geneva, NY and Salem, OR. The F_1_ adults comprised an equal or higher fraction than the F_2_ adults in 4 of 10 years in Geneva, NY (2008, 2009, 2013, and 2014) and 6 of 10 years in Salem, OR (2005, 2007, 2008, 2010, 2011, and 2012). A more detailed quantitative description of the range in F_1_ and F_2_ adults at the end of the year for each location is provided in Table 2.

**Figure 8:**
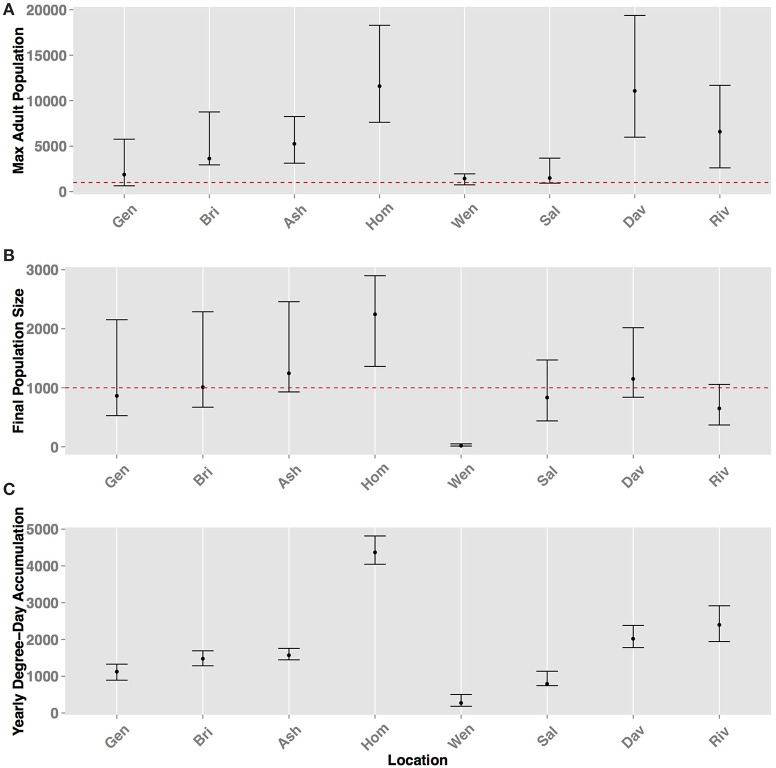
**Predicted population sizes (± range from all simulations and years) across all geographic locations for (A) maximum adult population size (B) final population size, and (C) yearly degree-day accumulation**. The error bars represent the standard errors from 100 simulations for the metrics.

There was significant (*p* < 0.01, using ANCOVA) variability of maximum population size and final population size by location (Figure 8). Yearly DD accumulation also served as an important covariate and there was a positive correlation (ρ > 0.8) between DD accumulation and maximum and final population size, with the only exception of Riverside, CA and Davis, CA, where Davis had higher population size despite lower yearly DD accumulation. This indicated that yearly DD accumulation was an important factor for predicting *H. halys* population sizes. Homestead, FL and Davis, CA had the highest mean maximum adult size in the 10-year period (both over 10,000 individuals) and not significantly different (*p* = 0.12) based on the Tukey's HSD test (Figure 8A). On average, positive population growth, (>1000 individuals), was seen in Asheville, NC, Homestead, FL and Davis, CA (Figure 8B). Other locations (Geneva, NY, Bridgetown, NJ, Salem, OR, and Riverside, CA) either had marginally positive population growth or marginal population declines. The range of final population sizes at all locations, however, encompassed a doubling or tripling in size, except in Wenatchee, WA. Wenatchee, WA is predicted to be unable to sustain population growth based on 10-year historical data (Figure 8B, Table 2). Wenatchee, WA also had the lowest accumulation in DD for *H. halys* development (Figure 8C, Figure S5).

## Discussion

We have developed an agent-based stage-specific model for the invasive *H. halys* that incorporates existing physiological information on development, survivorship and reproduction to predict population size, phenology and voltinism across a large geographic scale in the US. It is expected that this model could be applied to other insect species that share similar life history traits, specifically adult diapause and overlapping generations.

The model predicts bivoltinism potential across geographic locations but incorporation of the 13.5 h critical photoperiod has significant impacts on resulting population size across locations. This could influence the establishment and pest potential at locations where population growth was not positive (i.e., Wenatchee, WA). Despite a narrow window for development in the southern locations of Homestead, FL significant population growth is possible. Riverside, CA also has high DD accumulation but was predicted, on average, to not have sustainable population growth. This is likely due to the T_m_ threshold being reached, which is a limiting factor for temperate insect species. Research by Taylor et al. (2014) suggests that gut symbionts present on *H. halys* eggs also have a maximum temperature and exposure of eggs beyond T_m_ has impacts on hatch rate and fitness for multiple generations. Currently, this model could be used to redefine risk assessment scenarios and help guide management priorities throughout its invaded range and could be adapted to a web-interface for real-time predictions of phenological events for management purposes.

Population estimates based on visual observations and aggregation pheromone trap collections are highly variable and suggest *H. halys* is typically at low densities early in the spring (Nielsen and Hamilton, 2009; Leskey et al., 2012), which clouds the identification of a biofix and utility of DD models, and influenced our validation efforts. Our phenology model in Allentown, PA differed from observed estimates in the early season (May–June). This is likely due to difficulties finding widely dispersed clumped distributions of *H. halys* as they emerge from overwintering sites at low densities, including significant fractions in trees and human-built shelters (Rice et al., 2014). However, by mid and late season (July–October), the temporal patterns from predicted populations using a 13.5 h photoperiod biofix were well aligned with observed populations in all 3 years. Also, the model projected higher than average populations in Bridgeton, NJ in 2010 and 2011 (Figure 6, Figure S4), which aligns well with the dramatic increase in crop damage that occurred in the mid-Atlantic region in those years (Leskey et al., 2012).

A 13.5 h photoperiod is within, but at the lower end, of the reported range (Watanabe, 1979; Yanagi and Hagihara, 1980), and is consistent with insects that are in diapause during short-day conditions. Our current modeling efforts utilized a 13.5 h photoperiod for diapause termination and induction because it aligned well in the validation efforts, and provided a wider time scale for reproductive activity, resulting in the most conservative (e.g., highest) prediction for estimating potential population growth and voltinism at these temperate-climate locations. However, model predictions are clearly sensitive to assumptions about diapause termination in the spring, diapause induction after the summer equinox, and the maintenance of diapause in adults. Gray et al. (2001) effectively modeled diapause using two co-occurring temperature dependent processes, and it is highly probable that diapause in *H. halys* is influenced by multiple, and interacting, processes as opposed to the single process we utilized.

Model behavior was also strongly influenced by the assumption of the inability of vitellogenic adults to revert to a diapause state (a “lack of diapause plasticity”), and that this absence of diapause in vitellogenic adults resulted in death when temperatures dropped below freezing. In Wenatchee, WA, DD accumulation was the lowest among all sites, resulting in low population densities (Figure 8, Figure S5, note lower range in y-axis compared to other sites). In addition, the long interval between termination and initiation of diapause, due to the high latitude, was sufficient to enable temperature accumulation for development of F_2_ adults, most of which occurred prior to the critical photoperiod that would move them into diapause. Thus, these vitellogenic F_2_ adults were subject to the mortality rates applied to all adults, and killed by temperatures below 0°C. The lower densities coupled with the timing of F_2_ adults into vitellogenic status resulted in very low and possibly unsustainable populations by the end of the year (Figures 7, 8B). Many species have demonstrated plasticity in their response or rapid evolution to different critical photoperiods (Saunders et al., 1989). Although this has not been demonstrated for *H. halys*, the evolution of short-day response to photoperiod cues for diapause was demonstrated within a few decades of introduction for the invasive mosquito *Aedes albopictus* in the US and is an important adaptation to climatic variation in its invaded range (Urbanski et al., 2012). Pitcher-plant mosquitoes, *Wyeomyia smithii, also* demonstrate plasticity in photoperiodic response under climate change scenarios with a more pronounced shift in critical photoperiods in northern latitudes (Bradshaw and Holzapfel, 2001). An additional important assumption of the *H. halys* model is that the diapause cues are consistent across geographic locations, which is in agreement with the small founding population of the Eastern US population but these cues are under significant selection pressure (Xia et al., 2012; Xu et al., 2014). Thus, if *H. halys* evolves a differential response to photoperiod as seen with *A. albopictus*, or haplotypic diversity that results in varying diapause characteristics, the model predictions about population size and growth would change significantly and may expand its range and pest potential.

Agent-based approaches could further refine CLIMEX-based distribution models. Zhu et al. (2012) estimated a disjunct establishment probability of *H. halys* in the US with higher establishment probabilities in the mid-Atlantic, extending into the mid-West and upper South, and the West Coast. This assumes that the existing US populations carry the adaptive potential expressed throughout the geographic range of the insect in Asia. While our phenology model predicts very limited population growth in most years in areas such as Wenatchee, WA and Geneva, NY, it does predict large population growth in more southern locations such as Davis, CA and Homestead, FL, which is in conflict with the climate-matching model. Current populations in Davis, CA anecdotally support our model's prediction and were estimated by the application of additional parameters by Zhu et al. (2012).

Our model has many implications for prioritizing management. Management of *H. halys*, as in other Pentatomids, is difficult due to extensive movement among hosts, landscape elements, and difficulties in field estimates of population densities (McPherson and McPherson, 2000; Nielsen and Hamilton, 2009; Wallner et al., 2014). Field sampled or modeled estimates for a field or orchard are less accurate or stable as individuals from other locations and overlapping life stages in the landscape immigrate or emigrate from that location. Our modeled predictions shows very strong overlap of multiple life stages, and generations, with the co-occurrence of P, F_1_, and F_2_ generation adults. However, the time span at which life stages overlapped, and degree of synchrony among years for a life stage, varied by location. Southern and warmer locations tended toward greater synchrony among years. For example, F_2_ eclosion spanned 2 days in Homestead, FL, compared to 21 days in Geneva, NY. This has implications for timing management directed at specific life stages, and for optimizing sampling efforts. Given the significant population growth observed at most sites, it also emphasizes the importance of managing the P and F_1_ generations Figures S6, S7. Although populations were bivoltine at all locations, simply defining the voltinism potential at a location as a fixed number becomes confounding and can be misleading. In some locations and years (for example Salem, OR) F_2_ adults occurred, but at very low numbers, and it was F_1_ adults that contributed most strongly to the population at the end of the season. Insect management programs that aim to reduce region-wide populations by targeting overwintering adults, as proposed by Cross (1973) and later accomplished for the boll weevil eradication program, could start much earlier in locations where F_1_ adults were a significant fraction of the overwintering population. Field estimates are not able to distinguish among P and F_x_ adults, thus a modeling framework that effectively expresses distributions of all life stages becomes useful for management.

Importantly, this model does not address all factors that influence insect population phenology or dynamics. We present this model as a synthesis of current understanding of primarily abiotic factors (temperature, photoperiod) influencing *H. halys* phenology and potential population growth. However, our approach aligns well with field observations in Allentown, PA and suggests that our model has sufficient realism to warrant extrapolation to other locations and years. The agent-based approach we utilized could be applied to other insect species with overlapping generations and which diapause in the adult or other life stages. Recently, agent-based modeling has been applied to a few insect models, and phenology models have been applied to consider effects of climate change. Here, we extend the application to addressing invasive species. We present results under current climate conditions, and the model can be readily adapted to extrapolate to future climate scenarios.

## Author contributions

All three authors contributed equally to the formulation of the ideas, review of data and the analysis. SF initiated this project. SC conducted the modeling based off of parameters and data derived from AN with input and interpretation by SF. Both AN and SF contributed to evaluation of model outputs and decisions about physiological characteristics. All three authors contributed to drafting and editing the manuscript. This is a truly collaborative effort.

### Conflict of interest statement

The authors declare that the research was conducted in the absence of any commercial or financial relationships that could be construed as a potential conflict of interest.

## Supplementary material

Figure S1 **Simulated population size and phenology of adults transitioning out of reproductive diapause in the Spring and into reproductive diapause in the Fall in Allentown, PA, 2007**. Simulation initiation with 1000 overwintered parental adults.

Figure S2 **Predicted total population size by life Stage for the Eastern US (A) Geneva, NY, (B) Bridgeton, NJ, (C) Asheville, NC, (D) Homestead, FL, from 2005 through 2014**. Populations were initialized with 1000 adults for each year and simulation run.

Figure S3 **Predicted total population size by life Stage for the Western US (A) Wenatchee, WA, (B) Salem, OR, (C) Davis, CA, (D) Riverside, CA from 2005 through 2014**. Populations were initialized with 1000 adults for each year and simulation run.

Figure S4 **Degree-day accumulation for *Halyomorpha halys* development in the Eastern US (A) Geneva, NY, (B) Bridgeton, NJ, (C) Asheville, NC, (D) Homestead, FL, from 2005 through 2014**.

Figure S5 **Degree-day accumulation for *Halyomorpha halys* development in the Western US (A) Wenatchee, WA, (B) Salem, OR, (C) Davis, CA, (D) Riverside, CA from 2005 through 2014**.

Figure S6 **Predicted total population size by generation for the Eastern US (A) Geneva, NY, (B) Bridgeton, NJ, (C) Asheville, NC, (D) Homestead, FL, from 2005 through 2014**.

Figure S7 **Predicted total population size by generation for the Western US (A) Wenatchee, WA, (B) Salem, OR, (C) Davis, CA, (D) Riverside, CA from 2005 through 2014**.
