# Corrigendum: Coupling Developmental Physiology, Photoperiod, and Temperature to Model Phenology and Dynamics of an Invasive Heteropteran, *Halyomorpha halys*

**DOI:** 10.3389/fphys.2017.00568

**Published:** 2017-08-11

**Authors:** Anne L. Nielsen, Shi Chen, Shelby J. Fleischer

**Affiliations:** ^1^Department of Entomology, Rutgers University Bridgeton, NJ, United States; ^2^Department of Population Health and Pathobiology, North Carolina State University Charlotte, NC, United States; ^3^Department of Entomology, Pennsylvania State University University Park, PA, United States

**Keywords:** brown marmorated stink bug, phenology, agent-based model, stochastic model, life-history, population dynamics, invasive species

In this manuscript, we present model results from eight locations over 10 years based on temperatures at weather stations and photoperiod. We inadvertently made a poor choice for a weather station to represent Wenatchee WA. To avoid heat island effects, we chose the Grouse Camp weather station to represent Wenatchee. However, although Grouse Camp is only 21 km from Wenatchee, it is in a mountainous area (1,642 m elevation) and poorly represents the climate in the apple growing region of Wenatchee, WA (190 m elevation). Thus, the results do not reflect potential population dynamics of *H. halys* in Wenatchee. In re-evaluation of the model, the data show that populations at Wenatchee, WA, are predicted to behave similarly to those at Salem, OR, with an average albeit marginal positive population growth. Conclusions that were driven heavily by photoperiod, such as the range in days for initiation of oviposition by overwintered adults, were less affected (from 16 down to 13 days). The strong differences were due to markedly higher degree day accumulations at Wenatchee versus Grouse Camp.

The following six files use the same order of tables and figures from the original manuscript and use the Weather Station data for Wenatchee, WA (network ID: GHCND:USC00459074).

- Table 2. Model outputs defining key population parameters for the years 2005–2014.
- Figure 7. Model predictions of adult population size for Wenatchee, WA. *P* represented parental overwintered adults, which was initialized as 1000 for each year and for each simulation run.
- Figure 8. Predicted population sizes (+/− range from all simulations and years) across all geographic locations for **(A)** maximum adult population size, and **(B)** final population size, and **(C)** yearly degree-day accumulation. The error bars represent the standard errors from 100 simulations for the metrics.
- Figure S3. Predicted total population size by life stage for Wenatchee, WA from 2005 through 2014. Populations were initialized with 1000 adults for each year and simulation run.
- Figure S5. Degree-day accumulation for *Halyomorpha halys* development in Wenatchee, WA from 2005 through 2014.
- Figure S7. Predicted total population size by generation for Wenatchee, WA.

**Table 2:** Model outputs defining key population parameters for the years 2005-2014.

**Location**	**Coordinates**	**Crop**	**Non-diapause range**	**P Oviposition**		**F**_**1**_ **Eclosion**		**F**_**2**_ **Eclosion**		**Final adult population**	
				**Range**	**Median**	**Range**	**Median**	**Range**	**Median**	**F_1_**	**F_2_**
Geneva NY	42.88°N 76.99°W	Apple	Apr 18–Aug 26	May 30–Jun 17	Jun 7	Jun 21–Jul 18	Jun 30	Jul 19–Aug 9	Jul 28	243–447	81–1,847
Bridgeton NJ	39.43°N 75.23°W	Peach/Vegetable	Apr 22–Aug 22	Jun 3–Jun 6	Jun 4	Jun 9–Jul 2	Jun 23	Jul 6–Jul 31	Jul 24	140–278	531–2,027
Asheville NC	35.58°N 85.56°W	Tree fruit/Vegetable	Apr 28–Aug 17	May 28–May 29	May 29	Jun 3–Jun 11	Jun 6	Jun 25–Jul 4	Jul 2	89–253	803–2,287
Homestead FL	25.47°N 80.47°W	Tomato/Strawberry	May 24–July 22	Jun 5–Jun 6	Jun 6	Jun 17–Jun 18	Jun 18	Jul 8–Jul 10	Jul 9	44–137	1318–2,781
Wenatchee WA	47.42°N 120.33°W	Apple/Pear	Apr 14–Aug 31	Jun 2–Jun 15	Jun 8	Jun 17–Jun 29	Jun 22	Aug 1–Aug 18	Aug 6	106–559	46–1,557
Salem OR	44.93°N 123.03°W	Tree fruit/Wine grape	Apr 17–Aug 28	Jun 8–Jun 9	Jun 9	Jun 17–Jul 2	Jun 26	Jul 24–Jul 31	Jul 27	269–716	33–1,035
Davis CA	38.55°N 121.74°W	Tomato	Apr 24–Aug 20	Jun 3–Jun 4	Jun 4	Jun 14–Jun 19	Jun 16	Jul 6–Jul 15	Jul 11	75–255	733–1,893
Riverside CA	33.95°N 117.40°W	Citrus	May 1–Aug 13	Jun 10–Jun 11	Jun 11	Jun 20–Jun 24	Jun 23	Jul 8–Jul 16	Jul 13	21–95	349–962

**Figure 7:**
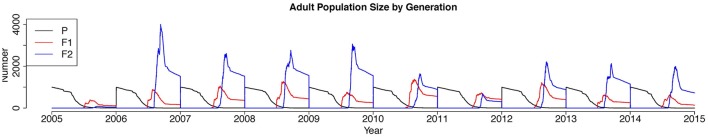
Model predictions of adult population size for Wenatchee, WA. *P* represented parental overwintered adults, which was initialized as 1,000 for each year and for each simulation run.

**Figure 8:**
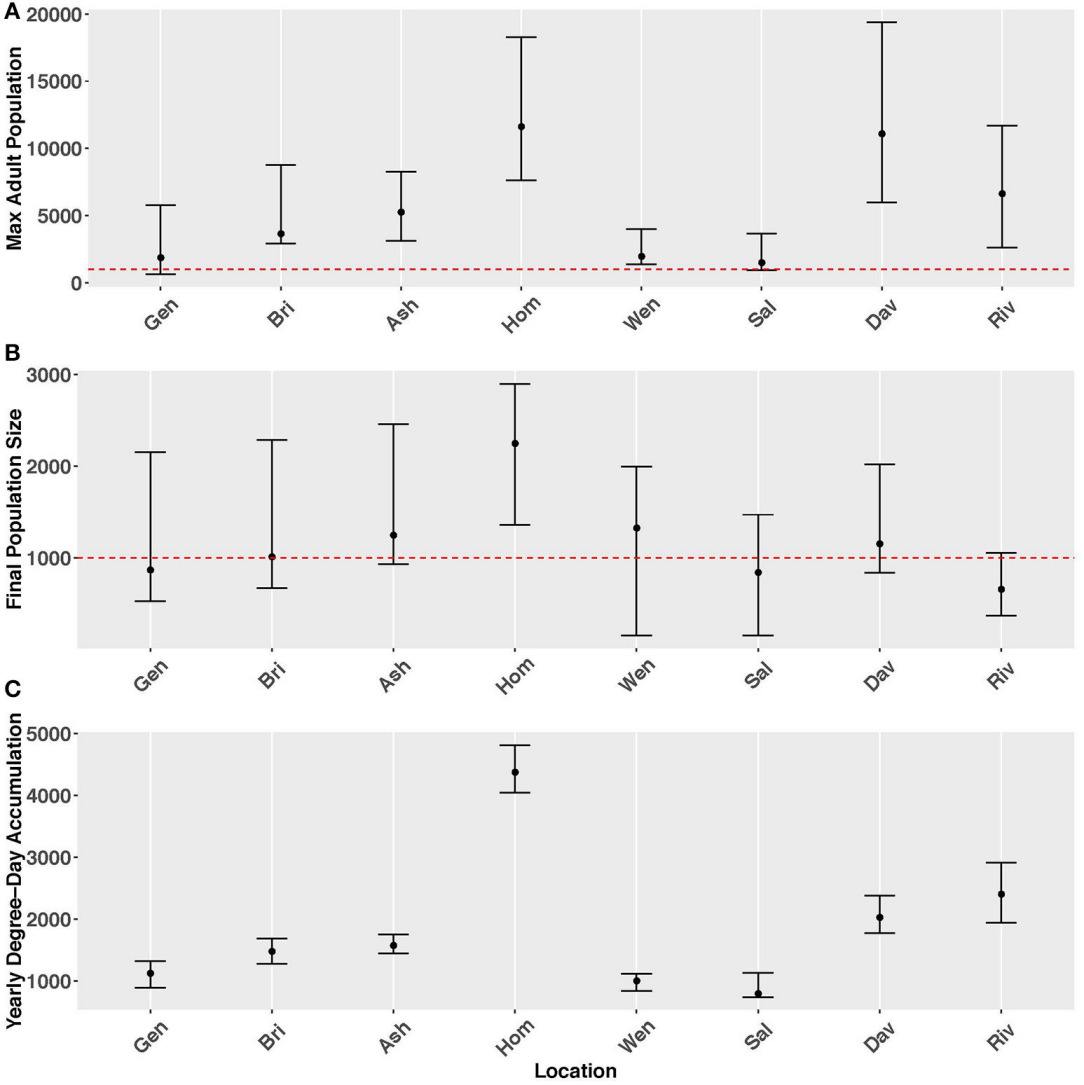
Predicted population sizes (+/− range from all simulations and years) across all geographic locations for **(A)** maximum adult population size, and **(B)** final population size, and **(C)** yearly degree-day accumulation. The error bars represent the standard errors from 100 simulations for the metrics.

We thank V. Jones for bringing this error to our attention, and the Frontiers journal for allowing us to add this Corrigendum.

## Conflict of interest statement

The authors declare that the research was conducted in the absence of any commercial or financial relationships that could be construed as a potential conflict of interest.
