# Supplementary figures and images for: Coupling Developmental Physiology, Photoperiod, and Temperature to Model Phenology and Dynamics of an Invasive Heteropteran, Halyomorpha halys
Source: Front Physiol. 2016 May 18;7:165. doi: 10.3389/fphys.2016.00165 (PMC4870838; doi:10.3389/fphys.2016.00165)

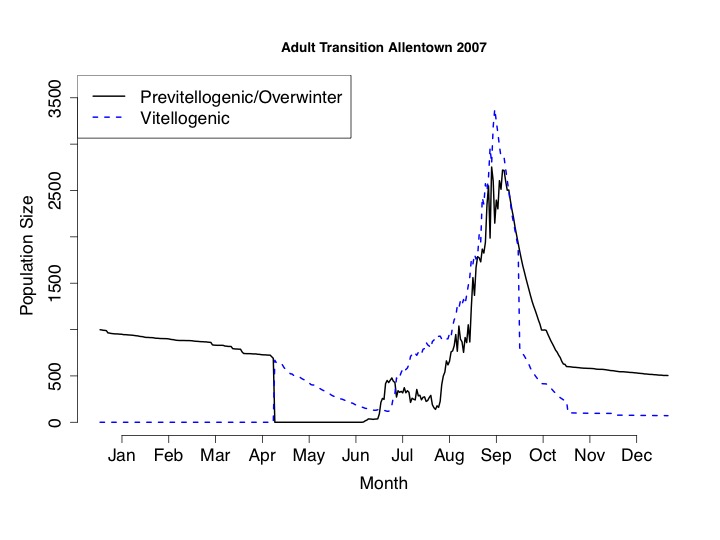

Supplement: Figure S1 — Simulated population size and phenology of adults transitioning out of reproductive diapause in the Spring and into reproductive diapause in the Fall in Allentown, PA, 2007. Simulation initiation with 1000 overwintered parental adults. [file Image1.jpeg]

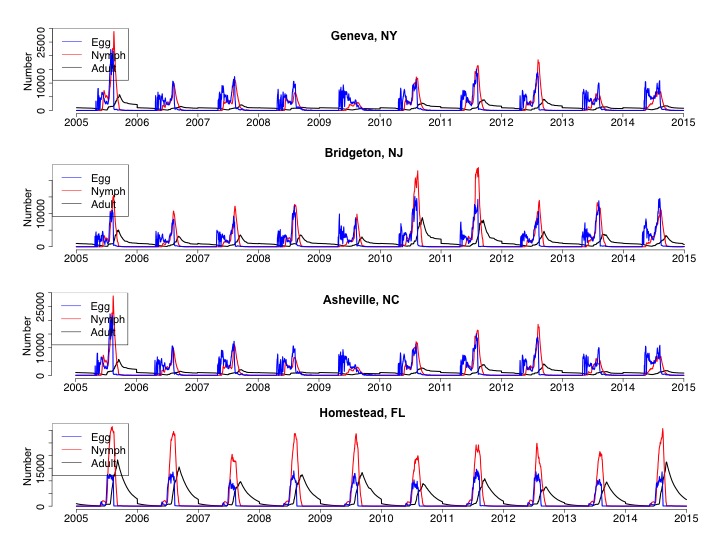

Supplement: Figure S2 — Predicted total population size by life Stage for the Eastern US (A) Geneva, NY, (B) Bridgeton, NJ, (C) Asheville, NC, (D) Homestead, FL, from 2005 through 2014. Populations were initialized with 1000 adults for each year and simulation run. [file Image2.jpeg]

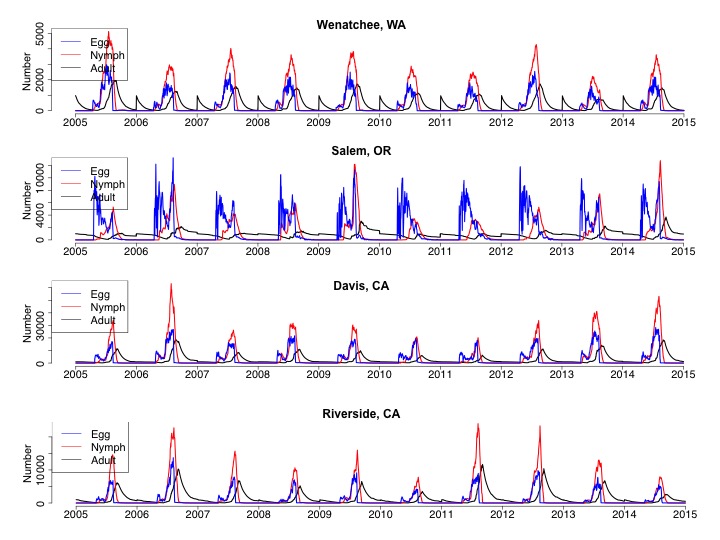

Supplement: Figure S3 — Predicted total population size by life Stage for the Western US (A) Wenatchee, WA, (B) Salem, OR, (C) Davis, CA, (D) Riverside, CA from 2005 through 2014. Populations were initialized with 1000 adults for each year and simulation run. [file Image3.jpeg]

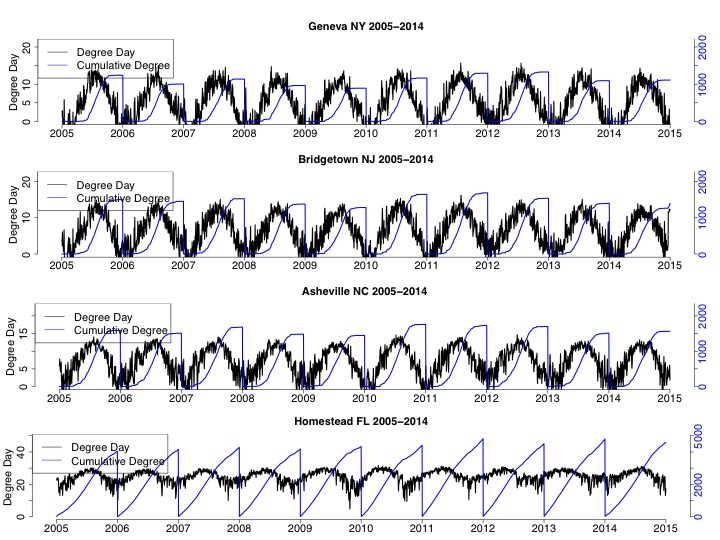

Supplement: Figure S4 — Degree-day accumulation for Halyomorpha halys development in the Eastern US (A) Geneva, NY, (B) Bridgeton, NJ, (C) Asheville, NC, (D) Homestead, FL, from 2005 through 2014. [file Image4.jpeg]

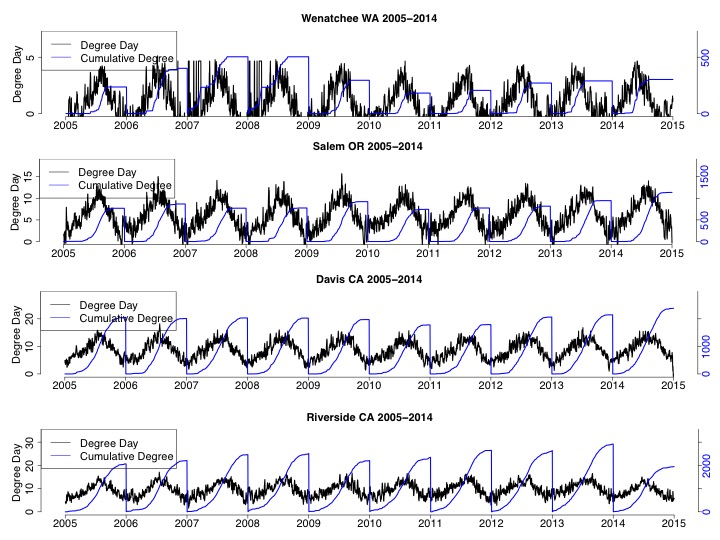

Supplement: Figure S5 — Degree-day accumulation for Halyomorpha halys development in the Western US (A) Wenatchee, WA, (B) Salem, OR, (C) Davis, CA, (D) Riverside, CA from 2005 through 2014. [file Image5.jpeg]

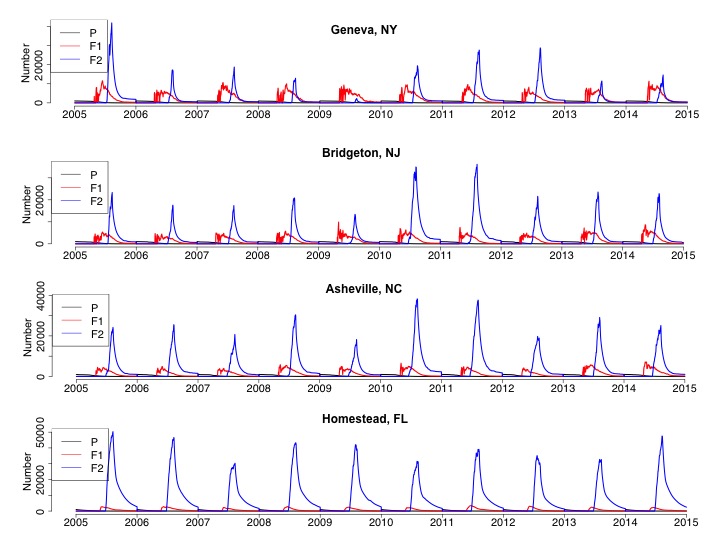

Supplement: Figure S6 — Predicted total population size by generation for the Eastern US (A) Geneva, NY, (B) Bridgeton, NJ, (C) Asheville, NC, (D) Homestead, FL, from 2005 through 2014. [file Image6.jpeg]

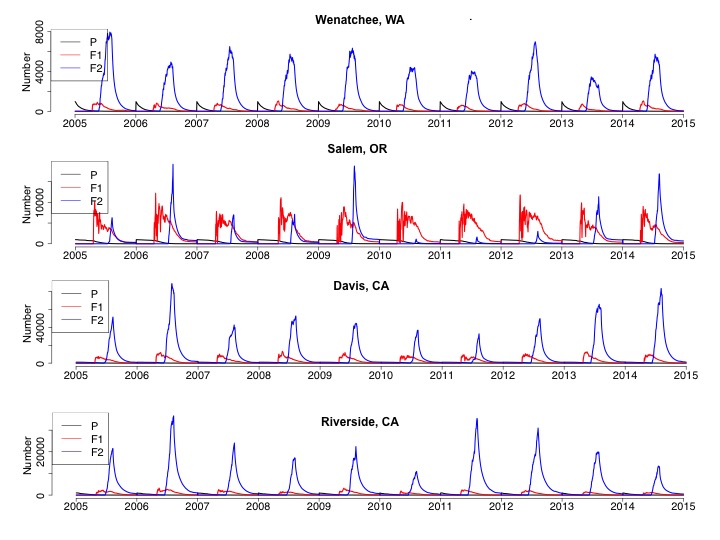

Supplement: Figure S7 — Predicted total population size by generation for the Western US (A) Wenatchee, WA, (B) Salem, OR, (C) Davis, CA, (D) Riverside, CA from 2005 through 2014. [file Image7.jpeg]
